# Quality Assurance Considerations in Radiopharmaceutical Therapy Dosimetry Using PLANETDose: An International Atomic Energy Agency Study

**DOI:** 10.2967/jnumed.122.265340

**Published:** 2024-01

**Authors:** Gunjan Kayal, Nathaly Barbosa, Carlos Calderón Marín, Ludovic Ferrer, José-Alejandro Fragoso-Negrín, Darko Grosev, Santosh Kumar Gupta, Nur Rahmah Hidayati, Tumelo C.G. Moalosi, Gian Luca Poli, Parul Thakral, Virginia Tsapaki, Sébastien Vauclin, Alex Vergara-Gil, Peter Knoll, Robert F. Hobbs, Manuel Bardiès

**Affiliations:** 1 CRCT, UMR 1037, INSERM, Université Toulouse III Paul Sabatier, Toulouse, France;; 2 SCK CEN, Belgian Nuclear Research Centre, Mol, Belgium;; 3 Instituto Nacional de Cancerología, Bogotá, Colombia;; 4 Instituto de Oncología y Radiobiología, Havana, Cuba;; 5 Medical Physics Department, ICO René Gauducheau, Nantes, France;; 6 CRCINA, UMR 1232, INSERM, France;; 7 DOSIsoft SA, Cachan, France;; 8 IRCM, UMR 1194 INSERM, Universite de Montpellier and Institut Regional du Cancer de Montpellier, Montpellier, France;; 9 Department of Nuclear Medicine and Radiation Protection, University Hospital Centre Zagreb, Zagreb, Croatia;; 10 Department of Nuclear Medicine and PET, Mahamana Pandit Madanmohan Malviya Cancer Centre and Homi Bhabha Cancer Centre, Varanasi, India;; 11 Research Center and Technology for Radiation Safety and Metrology–National Research and Innovation Agency, Jakarta, Indonesia;; 12 Department of Medical Imaging and Clinical Oncology, Medical Physics, Nuclear Medicine Division, Faculty of Medicine and Health Science, Stellenbosch University, Tygerberg Hospital, Cape Town, South Africa;; 13 Department of Medical Physics, ASST Papa Giovanni XXIII, Bergamo, Italy;; 14 Department of Nuclear Medicine, Fortis Memorial Research Institute, Gurugram, India;; 15 Dosimetry and Medical Radiation Physics, International Atomic Energy Agency, Vienna, Austria;; 16 Johns Hopkins Medical Institute, Baltimore, Maryland; and; 17 Département de Médecine Nucléaire, Institut Régional du Cancer de Montpellier, Montpellier, France

**Keywords:** clinical dosimetry, SPECT/CT, quality assurance, PLANETDose, variability assessment

## Abstract

Implementation of radiopharmaceutical therapy dosimetry varies depending on the clinical application, dosimetry protocol, software, and ultimately the operator. Assessing clinical dosimetry accuracy and precision is therefore a challenging task. This work emphasizes some pitfalls encountered during a structured analysis, performed on a single-patient dataset consisting of SPECT/CT images by various participants using a standard protocol and clinically approved commercial software. **Methods:** The clinical dataset consisted of the dosimetric study of a patient administered with [^177^Lu]Lu-DOTATATE at Tygerberg Hospital, South Africa, as a part of International Atomic Energy Agency–coordinated research project E23005. SPECT/CT images were acquired at 5 time points postinjection. Patient and calibration images were reconstructed on a workstation, and a calibration factor of 122.6 Bq/count was derived independently and provided to the participants. A standard dosimetric protocol was defined, and PLANETDose (version 3.1.1) software was installed at 9 centers to perform the dosimetry of 3 treatment cycles. The protocol included rigid image registration, segmentation (semimanual for organs, activity threshold for tumors), and dose voxel kernel convolution of activity followed by absorbed dose (AD) rate integration to obtain the ADs. Iterations of the protocol were performed by participants individually and within collective training, the results of which were analyzed for dosimetric variability, as well as for quality assurance and error analysis. Intermediary checkpoints were developed to understand possible sources of variation and to differentiate user error from legitimate user variability. **Results:** Initial dosimetric results for organs (liver and kidneys) and lesions showed considerable interoperator variability. Not only was the generation of intermediate checkpoints such as total counts, volumes, and activity required, but also activity-to-count ratio, activity concentration, and AD rate-to-activity concentration ratio to determine the source of variability. **Conclusion:** When the same patient dataset was analyzed using the same dosimetry procedure and software, significant disparities were observed in the results despite multiple sessions of training and feedback. Variations due to human error could be minimized or avoided by performing intensive training sessions, establishing intermediate checkpoints, conducting sanity checks, and cross-validating results across physicists or with standardized datasets. This finding promotes the development of quality assurance in clinical dosimetry.

## Visual Abstract

**Figure:**
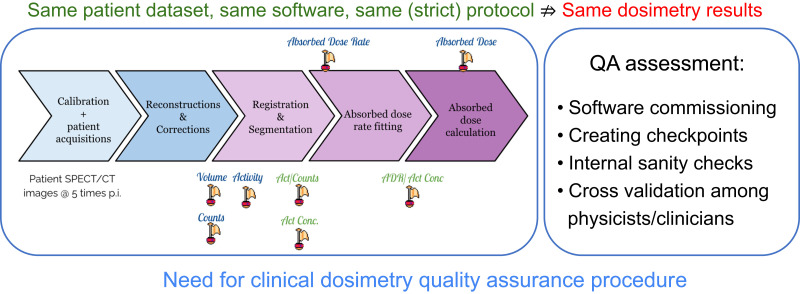

Radiopharmaceutical therapy (RPT) is based on the administration of radiolabeled vectors designed to concentrate cytotoxic levels of radiation in targets while preserving the surrounding healthy tissues. In comparison to external-beam radiation therapy, which involves personalized treatment regimens, most RPT administers a fixed activity. For example, the European Medicines Agency and the Food and Drug Administration approved [^177^Lu]Lu-DOTATATE (Lutathera; Novartis) for treatment of neuroendocrine tumors as four 7.4-GBq injections separated by 8-wk intervals (1*,*^2^).

A patient-specific treatment approach would allow a major paradigm shift from the one*-*size-fits-all approach to personalized medicine in which the optimal activity is specifically assessed for each patient. In article 56 (Optimization) (3), Euratom Directive 2013/59 (applicable within the European Union) requests that “For all medical exposure of patients for radiotherapeutic purposes, exposures of target volumes shall be individually planned, and their delivery appropriately verified taking into account that doses to non-target volumes and tissues shall be as low as reasonably achievable and consistent with the intended radiotherapeutic purpose of the exposure.” Even though personalized planning cannot be achieved with fixed activities, the verification of irradiation delivered can always be assessed. Also, several authors (4–7) have advocated the feasibility of patient-specific RPT dosimetry. Evidence of the absorbed dose (AD)–effect relationship has been published in several clinical indications (8–11).

The clinical dosimetry workflow consists of several steps, from radiopharmaceutical pharmacokinetics determination in the volumes of interest (VOIs), most often through sequential quantitative imaging, to AD or other standardized dosimetry quantity computation, such as biologic effective dose or equieffective dose (12). Currently, RPT dosimetry either is not widely implemented in clinical facilities (13–15) or, if implemented, may differ in objectives and sophistication among centers, thereby resulting in a large variability of dosimetric approaches and results (16).

However, there are 2 facets of variability. Clinical choices that govern dosimetric approaches induce natural variability and should not preclude the use of personalized dosimetry. More concerning is the potential variability of results for a given clinical application, due to different methodologies or even to general lack of expertise. It is therefore desirable to be able to compare results obtained in different clinical centers for a given therapeutic approach.

The Society of Nuclear Medicine and Molecular Imaging presented the preliminary results of a study aiming to standardize and harmonize dosimetry procedures by assessing the variability introduced in various dosimetry steps. The study revealed large differences in time-integrated activity (TIA) and in ADs. The participants in this study chose their own methodologies for VOI delineation, TIA integration, TIA dosimetry, and reported volumes, TIA, AD rates (ADRs), and AD; however, neither the sources of variation nor the origins of the outliers were obvious (17). Subsequent detailed analysis (18) demonstrated that there were transcription, methodologic, and reporting errors and differences in methods and decisions that contributed substantially to the large variations. In this analysis (18) for pure SPECT data, removing variabilities due to errors resulted in quartile coefficients of dispersion of 10%–30% in the organs and 10%–40% in the lesions for the 2 patients used in the study. Variabilities were further reduced when participants were given VOIs or TIAs.

In 2017, the International Atomic Energy Agency initiated coordinated research project (CRP) E2.30.05, “Dosimetry in Radiopharmaceutical Therapy for Personalized Patient Treatment,” to educate and train volunteer medical physicists and implement harmonized dosimetric procedures, along with assessing the global accuracy of RPT dosimetry. This was done by conducting multiple training sessions and iterations using a clinical SPECT/CT patient dataset.

Consequently, the ground truth activity and ADs of the dataset are not known and the study cannot assess for accuracy. The goal of this work is to assess variability or precision of results and to identify and eliminate methodologic errors that increase variability as part of an overall educational goal.

## MATERIALS AND METHODS

### Participants

The participating institutions were from Colombia, Croatia, Cuba, France, India, Indonesia, South Africa, and the United States. Each performed dosimetry with its respective expertise and the specific training acquired throughout this work. The selection of institutions was based on International Atomic Energy Agency reviews of proposals to join the CRP, except for the United States and France, whose participation was based on their substantial experience in this field.

### Clinical Dataset

The clinical dataset was derived from the dosimetric study of a [^177^Lu]Lu-DOTATATE patient from Tygerberg Hospital, South Africa. Activities of 6.24, 6.67, 6.85, and 6.03 GBq were administered in a first, second, third, and fourth therapy cycle, respectively, with an interval of 11 wk between cycles. Patient SPECT/CT images were acquired at 5 time points after injection (1–2, 4, 24, 48, and 96 h) on a GE Healthcare Infinia Hawkeye 4 (9.5-mm [^3^/_8_-in] NaI crystal thickness and medium-energy collimator) with calibration images and reconstructed on a Hermes Medical Solutions (version 4.15) workstation. A calibration phantom was imaged using the same acquisition and reconstruction settings. All parameters were specified by Kayal et al. (19). A calibration factor of 122.6 Bq/count (or 4.53 cps/MBq with 1,800 s of acquisition time) was obtained and provided to each center.

### Definition of Standard Protocol

A standard dosimetric protocol was defined, and PLANETDose (version 3.1.1) from DOSIsoft SA was used for the first 3 therapy cycles. The fourth cycle was not examined because there was no adequate SPECT/CT imaging. Since reconstructed patient images were circulated to each center, the common dosimetry protocol was defined beginning at the registration step. All steps of the protocol were followed by all participants independently on their workstations.

#### Registration and Segmentation

Automatic rigid registration for all time points was performed by each site considering the first CT scan as the reference. The segmented VOIs included lesions (anterior, lateral, posterior, and inferior) along with both kidneys and whole and healthy liver. Each participating center contoured the normal organs (kidneys and liver) semimanually on the first CT scan with an interpolation process and then propagated in a rigid way through all registered CT scans while performing lesion segmentation on SPECT images at each time point using a 40% threshold of the maximal uptake. Afterward, a 4-dimensional exclusion Boolean operation between the anatomic liver (or whole liver) and the 4 lesions gave the contour of normal liver.

#### ADR and AD

ADRs were obtained for each VOI and cycle using the dose voxel kernel convolution algorithm with density correction within PLANETDose. ADs were obtained by integrating ADR over time. Each participant selected a fit among mono-, bi-, or triexponential fitting or trapezoidal fitting, on the basis of the participant’s expertise. The fit functions and parameters used by each participant can be provided on request.

#### Data Exportation

For each VOI, the volume, total counts, and activity per time point were exported. The values for normal organs and lesions were taken from the anatomic and functional contouring result tables, respectively (PLANETDose uses a dual anatomic/functional mode system). The fitting parameters (fitting equation and *R*^2^) along with the ADs in each VOI were generated and stored.

### Statistics

To quantify the variations among participants, the mean and median along with the associated uncertainties were computed using previously published equations (20). The *t*-test was performed to examine the significance of the difference between the fitting techniques used among participants (*P* ≤ 0.05) (21*,*^22^).

### Optimization Procedure

The CRP project aimed to contribute to the standardization of RPT dosimetry and help participants develop and implement harmonized dosimetric procedures.

During each brainstorming session, the results were discussed, and further training was provided to the participants, resulting in an iteration of the dosimetry calculations. The number of iterations for the first, second, and third therapy cycles was 1–4, 2–3, and 4 or more, respectively. Even though the results for each center were not available to all, the procedure was not a masked intercomparison but rather evolved to an elaborate training procedure, with the objective of ensuring proper dosimetric understanding and application while allowing for limited individual choices in registration, contouring, and integration.

**FIGURE 1.**
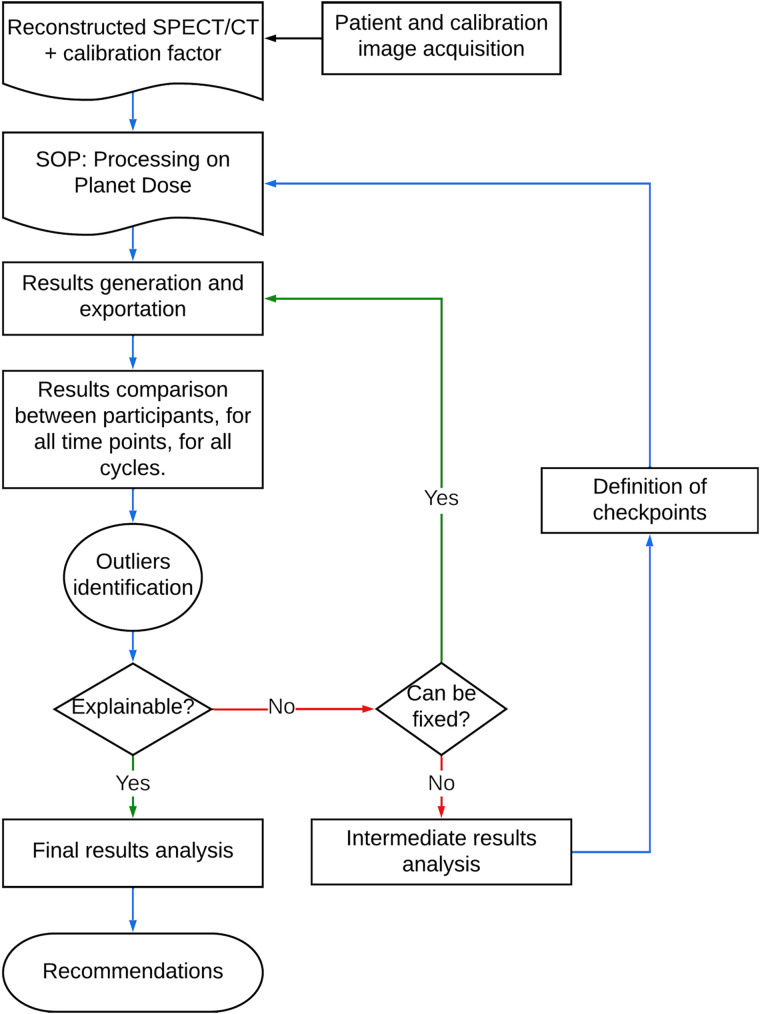
Workflow used for analyzing data and computing final results and recommendations. SOP = standard operating procedure.

The identification of outlier results led participants to evaluate the integrity of their results as shown in Figure 1. Other variations observed consistently throughout the data necessitated the inclusion of additional quality assurance (QA) metrics (or checkpoints) in the dosimetric procedure with subsequent iterations of results. The results presented here are therefore the best that could be obtained within the time frame of the CRP, after extensive training and iterations.

## RESULTS

Results were obtained for each time point and VOI for the first 3 cycles. In this section, the salient results are highlighted as coefficients of variation of the median (CV_med_) among participants (ratio of median uncertainty to median); however, all results are available in Supplemental Tables 1 and 2 and Supplemental Figures 1–8 (supplemental materials are available at http://jnm.snmjournals.org).

### Activity Quantification

#### Volume Segmentation

**FIGURE 2.**
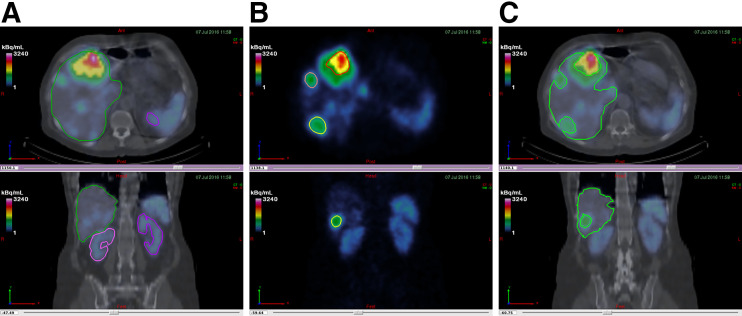
(A) Anatomic segmentation (for whole liver and kidneys). (B) Functional segmentation for lesions. (C) Normal liver from Boolean subtraction.

The liver and the kidneys along with the 4 lesions defined in the patient SPECT/CT images are shown in Figure 2 for the first cycle.

**FIGURE 3.**
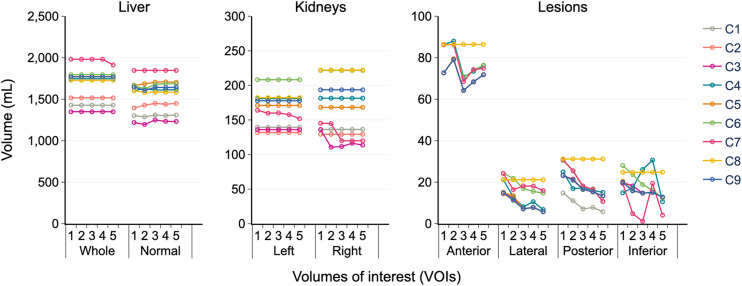
Organ and lesion volumes in first treatment cycle for 5 time points (1–5). Whole liver signifies anatomic liver, whereas normal liver represents healthy liver (whole liver − 4 lesions).

The volumes obtained by the 9 centers (C1–C9) for each VOI for the first cycle are plotted in Figure 3. The CV_med_ among time points and cycles ranged from 6% to 13% for normal organs and from 3% to 20% for lesions, with the smallest lesions exhibiting the largest variability. The variations in normal-organ volumes as a function of time at each center constitute evidence of error according to the propagation process defined in the protocol, since the volume—once defined on the first time point—is propagated to the other time points. Similarly, variations of the lesion volumes between time points may be expected, and each center, in principle, should have the same volume at each time point because the lesion volumes were defined using a predefined threshold (40% of maximum activity uptake relative to each time point). The range of volumes (along with the uncertainties) obtained by various participants for each VOI and each cycle is specified in Supplemental Table 1.

#### Derivation of Counts and Activity

**FIGURE 4.**
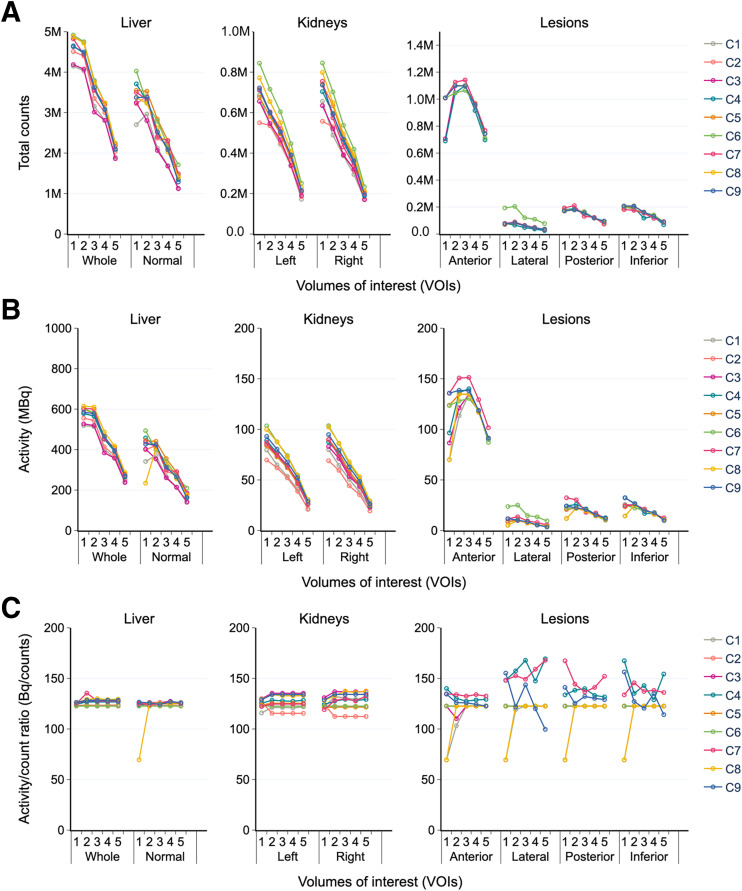
Total counts (A), activity (B), and activity-to-count ratio (C) for each VOI in third cycle for time points 1–5. Whole liver signifies anatomic liver, whereas normal liver represents healthy liver (whole liver − 4 lesions).

The total counts and activity in normal organs (kidneys, normal liver, and whole liver) and lesions are illustrated in Figures 4A and 4B for the third cycle. Counts and activities within each VOI tend to follow a similar trend for most time intervals and participants, although they are not identical as would be anticipated.

The whole liver demonstrated a significantly lower CV_med_ in volume and activity (∼5% and 3%–5%, respectively) at each time point and cycle analyzed. The lateral lesion had the widest range in volume (8%–59%) and activity (10%–49%). The other smaller lesions had a relatively lower activity (CV_med_, 2%–15%) comparable to that in the kidneys.

Outlier data are discernible. The counts and the activity for the lateral lesion were significantly higher at C6 than at the other centers (Fig. 4A) and can be attributed to this center’s larger lesion segmentation. In parallel, a significant activity decrease was seen in the lesions and the normal liver for the first time point for C8. A common trend in counts and activity was not systematically observed, thereby necessitating the generation of a new checkpoint activity-to-count ratio (Fig. 4C). The activity fluctuation for C8 might be attributed to incorrect calibration factor input or a software flaw linked with a particular time point, but these possibilities require further investigation.

### ADRs

**FIGURE 5.**
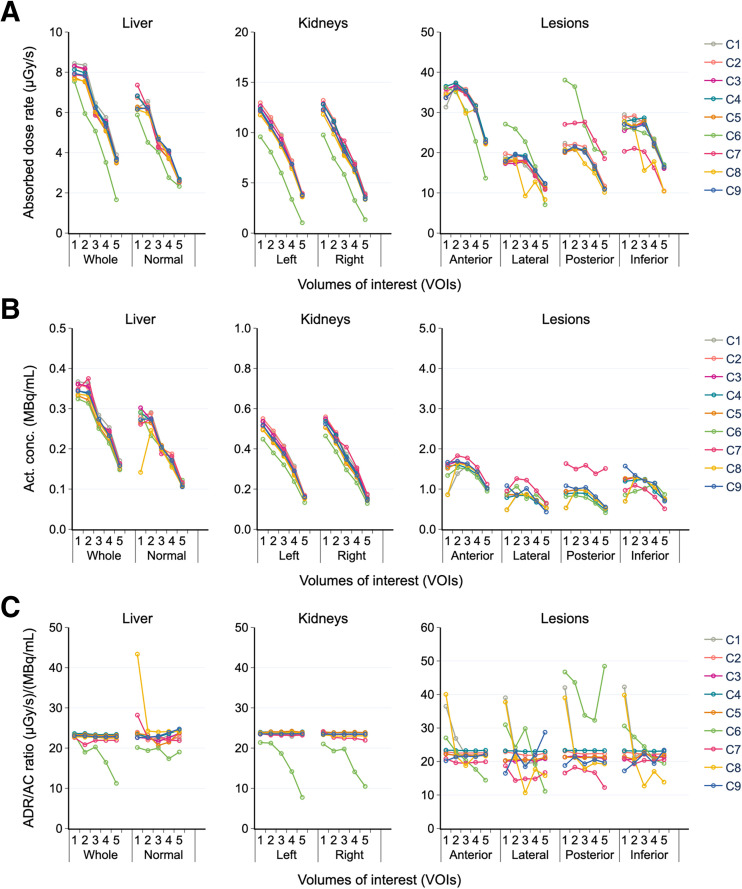
ADR (A), AC (B), and ADR-to-AC ratio (C) for each VOI in third cycle for time points 1–5. Whole liver signifies anatomic liver, whereas normal liver represents healthy liver (whole liver − 4 lesions).

Notable anomalies were observed for the third cycle (Fig. 5A). Except for results from C6, the ADR for time points for each organ was reasonably consistent between time points and participants. Unexpected ADR variability in lesions among participants initiated the generation of additional checkpoints: activity concentration (AC) and ADR-to-AC ratio. Since ADR is proportional to AC (for self-dose contributions to the AD dominating in regions of high uptake), it was hypothesized that the ADR-to-AC ratio would be relatively constant for each VOI.

Figure 5B shows the AC at each time point for the third cycle. All centers, including C6, had a fairly consistent AC for the liver and kidneys. Consequently, the ADR deviation of C6 in the liver and kidneys (6%–73%) cannot be directly related to volume segmentation or activity quantification but was most likely due to a transcriptional error. Additionally, the calculation of AC revealed that C8 likely overlooked some systematic error at the first time point.

The ADR-to-AC ratio (Fig. 5C) had nearly constant values (despite the presence of a few outliers) for the organs but not necessarily for the lesions. Because of the incoherent ADR and AC of C6, its ADR-to-AC ratio varied significantly. Variability in this ratio for lesions at the first time point for C8 is a consequence of error propagation from the activity quantification. Other variations were ascribed to transcriptional error.

### AD Calculations

**FIGURE 6.**
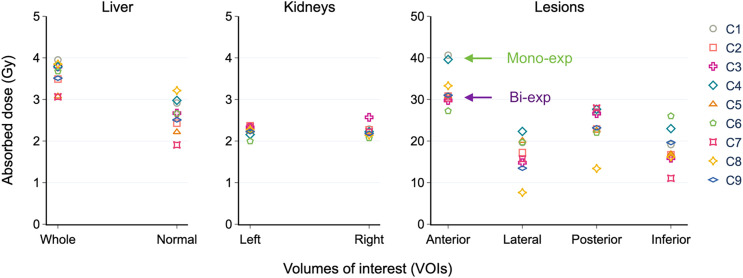
AD (in Gy) for each VOI (organs and lesions) in first cycle. Whole liver signifies anatomic liver, whereas normal liver represents healthy liver (whole liver − 4 lesions).

The ADRs were integrated using either monoexponential (corresponding to washout or decay curve) or biexponential (including one uptake and one washout phase) fitting models to obtain the ADs in each VOI. The fits chosen by centers for different VOIs are presented in Table 1. ADs for the first cycle are plotted in Figure 6. Low ADs (2–4 Gy) were obtained for the liver and kidneys, whereas ADs of up to 41 Gy were obtained for lesions. The AD obtained from monoexponential ADR fitting was substantially higher than that from biexponential ADR fitting (whole liver: 3.76 ± 0.15 Gy vs. 3.21 ± 0.24 Gy with *P* < 0.01; anterior lesion: 37.85 ± 3.98 Gy vs. 29.97 ± 1.42 Gy; *P* < 0.01).

**TABLE 1.** Fitting Chosen by Different Centers to Obtain AD for Each VOI

Treatment cycle		No. of centers	
	VOI	Monoexponential	Biexponential
	Organs	6	3
1	Anterior lesion	3	6
	Other lesions	1	8
	Organs	6	3
2	Anterior lesion*	3	5
	Other lesions	4	5
	Organs	6	3
3	Anterior lesion	3	6
	Other lesions	4	5

* Absence of fitting parameters from clinical center.

Although ADRs from one site (C6) varied by up to 73% for the third cycle, variability in organ ADs was generally acceptable across participants (Table 2). For lesions, several outliers in the AD were identified and most probably arose from the centers’ respective high or low ADR (C6 and C8). The high AD in the inferior lesion from C6 was not plausible given the reduced ADR and AC and was likely a consequence of a transcriptional error or mishandling of the software.

**TABLE 2.** AD in Each VOI for Each Treatment Cycle

VOI	Cycle 1	Cycle 2	Cycle 3
R kidney	2.24 ± 0.14	3.51 ± 0.29	3.33 ± 0.25
L kidney	2.25 ± 0.12	3.48 ± 0.18	3.50 ± 0.18
Whole liver	3.58 ± 0.33	3.87 ± 0.21	3.35 ± 0.17
Normal liver	2.61 ± 0.40	2.94 ± 0.28	2.28 ± 0.15
1, anterior	32.60 ± 4.56	34.68 ± 6.73	24.60 ± 2.00
2, lateral	16.73 ± 4.45	20.29 ± 6.40	12.22 ± 2.90
3, posterior	23.69 ± 4.53	28.57 ± 9.16	11.34 ± 3.75
4, inferior	18.27 ± 4.35	23.94 ± 6.36	15.77 ± 4.85

Data are mean ± SD (Gy).

Mean variations in the AD calculation for each cycle in each VOI can be seen in Table 2. The coefficient of variation in AD was up to 15% in the case of organs and up to 33% for the lesions.

## DISCUSSION

This work presents the dosimetry performed on patient image datasets acquired at different time points using PLANETDose software. Such a multicentric dosimetry comparison on a single clinical patient dataset using the same protocol and software by various centers appraises the precision of the dosimetry chain. The impact of reconstruction and calibration phases on the dosimetry chain was not considered in this work (23). The possibility of exporting intermediate results offered by PLANETDose facilitated the assessment of variations at each clinical dosimetry workflow step (24).

Although variations (CV_med_) in organ volumes ranged from 6% to 13% for normal organs, CV_med_ increased to 38% for lesions. Even though the conversion of counts to activity was merely a reflection of the calibration factor, it was remarkable to see higher variations in counts (3%–29% for normal organs and ≤46% for lesions) but not a similar trend in discrepancies in activity, particularly for normal organs (13%). The ADR varied by a maximum of 12% and 17%, respectively, for normal organs and lesions, whereas the integration of ADR to obtain ADs reduced the variation in normal organs (<8%) and increased it in lesions (≤33%).

**FIGURE 7.**
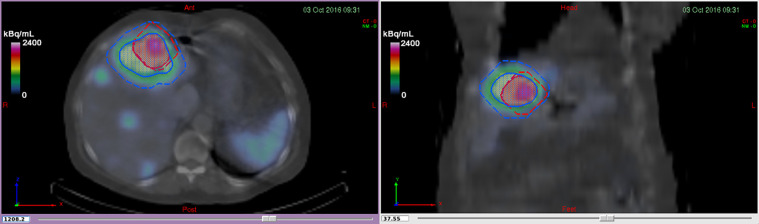
Typical error illustrating selection of small bounding box (in red) for anterior lesion while performing threshold-based segmentation. Blue VOI represents correct segmentation. *Bounding box* refers to predetermined subregion or selection in which thresholding is performed. By performing single click in area of high uptake, it is possible to generate tumor contour, thus preventing any potential correlation with uptake regions that are near tumor (e.g., organ, another tumor).

Every iteration evidenced significant disparities among participants, mostly derived from the heterogeneity of their initial level of expertise. To obtain more explainable results, these discrepancies were identified and addressed until the last iteration. Some of the observed issues were transcriptional error (improper unit conversions, use of a comma to represent decimals, errors in copying and pasting exported data), improper segmentation (inappropriate use of the thresholding technique, resulting in smaller segmented volumes; Fig. 7), and software mishandling (software data output formats were multiple and confusing, leading to incorrect reporting). However, since this was an educational and learning experience, most of these errors were deemed to be correctable.

Several additional checkpoints were defined during this dosimetric analysis: activity-to-count ratio, AC, and ADR-to-AC ratio. The 2 latter checkpoints could be incorporated in the software.

Even after compensation for obvious methodologic errors, the user-dependent fitting model generated significant variability. Providing users with additional leeway to choose their preferred methodologic dosimetric approach will probably lead to an even wider range of outcomes. This was seen in recently published data from Society of Nuclear Medicine and Molecular Imaging challenge users when allowed to choose the methodology (17) and when segmentation and TIA were provided (18).

The initial hypothesis that the same patient data, with the same processing workflow and using the same software, would yield relatively low interoperator variability proved to be false. Variable results were examined closely in peer review training sessions, the source of the discrepancy was searched for, and it was decided whether an avoidable error had been made, warranting a teaching moment, or whether the discrepancy was a justified user variation.

The large number of iterations needed to increase the proficiency of each user with the software highlighted the importance of training. Often, insufficient training is delivered to users after the acquisition of new software packages. Several practical training sessions (along with theory) could increase proficiency in clinical dosimetry. Also, having access to a benchmark clinical dataset that comes with a set of expected results, or at least a range of acceptable results, would be an invaluable asset of the training.

Best practices in RPT should be adopted to ensure that results are reliable, traceable, and reproducible—in other words, that they include robust QA. This comprises (but is not limited to) double processing using independent software (or free open-source tools) and cross-verification across physicists, as is current practice in external-beam radiation therapy. Because most clinical dosimetry software companies, even those with a long history in imaging, are relatively new to the field, a critical eye (and QA) on the procedure implemented by the user should also be accompanied by an equally critical use, including commissioning and regular QA of the software. In the present case, no commissioning or QA was performed initially, although several checks evolved to account for possible software inconsistencies (e.g., count-to-activity ratio).

Also, collaboration between physicists and physicians and between external-beam radiation therapy and RPT physicists would be a good starting point for implementing dosimetry QA since external-beam radiation therapy physicists are more experienced with error and failure mode analysis, therapeutic QA, and procedures. This requires a willingness to adapt to a new workplace culture of increasing cooperation (25).

It is vital to assess not only the precision but also the accuracy of the clinical dosimetry workflow. A possible way forward for this objective is the DosiTest project, in which simulated patient datasets (with activity being perfectly characterized at the voxel level for each time point) are used as the ground truth (26).

Finally, although not implemented here, using biologic effective dose or equieffective dose along with AD is an essential part of standardizing the dosimetric process and reporting (27*,*^28^).

## CONCLUSION

This paper illustrates how dosimetric analysis performed by various operators using the same protocol and software on one patient dataset may still result in large variations, as well as how practice and experience are needed after initial training to obtain reliable results in RPT dosimetry. Making the distinction between expected variability (related to legitimate operator choices) and erroneous processing (due to a variety of causes) was deemed crucial. The analysis of the results revealed the following important points.

### Software Commissioning

Use of software for calculation of AD and other dosimetric quantities requires a rigorous validation of the software itself, including checks on intermediate results and an end-to-end test.

### Checkpoints

The possibility to extract results at various intermediate steps of the clinical dosimetry workflow should be integrated, such as activity-to-count ratio or ADR-to-AC ratio.

### Sanity Checks

Internal checks implemented in the dosimetry package should minimize human mistakes: when obviously aberrant results are obtained, warning messages or even fatal errors should be generated.

### Result Validation

Most errors could be related to the fact that dosimetry was performed by single individuals. Cross-verification of results among physicists and clinicians should be systematically implemented, when possible.

This CRP enabled defining some aspects to include in a clinical dosimetry QA procedure. As such, it represents a stepping stone toward the definition of reliable and reproducible dosimetry procedures. This project also resulted in the creation of a benchmark dosimetry dataset that is adapted for training individuals in dosimetry. This should set a model for other clinical indications and software. The expected results and associated variability are now available for this patient dataset and procedure and will be the topic of further communication.

## Notes

KEY POINTS **QUESTION:** How can the sources of variation associated with clinical dosimetry be evaluated? **PERTINENT FINDINGS:** Dosimetry performed by several users on a single patient dataset with the same software and the same standard protocol resulted nonetheless in high variations and discrepancies among participants. The in-depth analysis required implementing checkpoints and internal sanity checks. **IMPLICATIONS FOR PATIENT CARE:** Implementing QA in clinical dosimetry starts with providing sufficient training and implementing cross validation of dosimetry results.
